# Electrical Impedance Tomography Predicts Weaning Success in Adult Patients With Delayed Upper Abdominal Surgery: A Single-Center Retrospective Study

**DOI:** 10.3389/fmed.2021.748493

**Published:** 2021-12-02

**Authors:** Jiajia Li, Fan Zeng, Fuxun Yang, Xiaoxiu Luo, Rongan Liu, Yinjie Ren, Yunping Lan, Yu Lei, Gaoping Zhao, Xiaobo Huang

**Affiliations:** ^1^Department of Intensive Care Unit, Sichuan Academy of Medical Sciences and Sichuan Provincial People's Hospital, Chengdu, China; ^2^Department of Gastrointestinal Surgery, Sichuan Academy of Medical Sciences and Sichuan Provincial People's Hospital, Chengdu, China

**Keywords:** success rate of weaning, upper abdominal surgery, electrical impedance tomography, mechanical ventilation, predict

## Abstract

**Objective:** To evaluate the predictive value of electrical impedance tomography (EIT) in patients with delayed ventilator withdrawal after upper abdominal surgery.

**Methods:** We retrospectively analyzed data of patients who were ventilated >24 h after upper abdominal surgery between January 2018 and August 2019. The patients were divided into successful (group S) and failed (group F) weaning groups. EIT recordings were obtained at 0, 5, 15, and 30 min of spontaneous breathing trials (SBTs) with SBT at 0 min set as baseline. We assessed the change in delta end-expiratory lung impedance and tidal volume ratio (ΔEELI/VT) from baseline, the change in compliance change percentage variation (|Δ(CW-CL)|) from baseline, the standard deviation of regional ventilation delay index (RVDSD), and global inhomogeneity (GI) using generalized estimation equation analyses. Receiver operating characteristic curve analyses were performed to evaluate the predictive value of parameters indicating weaning success.

**Results:** Among the 32 included patients, ventilation weaning was successful in 23 patients but failed in nine. Generalized estimation equation analysis showed that compared with group F, the ΔEELI/VT was lower, and the GI, RVDSD, and (|Δ(CW-CL)|) were higher in group S. For predicting withdrawal failure, the areas under the curve of the ΔEELI/VT, (|Δ(CW-CL)|), and the RVDSD were 0.819, 0.918, and 0.918, and 0.816, 0.884, and 0.918 at 15 and 30 min during the SBTs, respectively.

**Conclusion:** The electrical impedance tomography may predict the success rate of ventilator weaning in patients with delayed ventilator withdrawal after upper abdominal surgery.

## Introduction

Patients who are mechanically ventilated during upper abdominal surgery need to be weaned off of mechanical ventilation as soon as possible after they recover spontaneous breathing, which helps patients recover quickly. However, some patients experience varying degrees of post-operative lung function impairment because of reduced ventilatory muscle activity, diaphragmatic dysfunction, and decreased lung compliance (1). Older patients with preoperative lung disease or decreased lung function often require delayed extubation. The rapid shallow breathing index, occlusion pressure at 100 ms, and spontaneous breathing trials (SBTs) have been commonly used to predict weaning. However, the measured values of the rapid shallow breathing index and occlusion pressure at 100 ms can be inaccurate because of the patient's posture, airway stenosis, fever, and other factors. SBTs are commonly used in clinical practice, and its outcome is evaluated by objective parameters, such as the respiratory rate to tidal volume ratio (respiratory rate/VT) and arterial blood gases. Patients who pass the SBT can attempt ventilator weaning and endotracheal tube removal. However, some patients develop respiratory failure after weaning and need ventilator-assisted therapy. In patients requiring prolonged mechanical ventilation therapy, the rate of weaning failure is >10% (2). Failure to wean can increase the risk of mechanical ventilation complications and even increase the length of hospital stay and mortality. Therefore, it is necessary to identify a more specific way to evaluate the weaning process.

Electrical impedance tomography (EIT) is a new-generation, non-invasive functional imaging technology for real-time monitoring of pulmonary ventilation in patients. Based on different electrical impedances of tissues, gas, and liquid (which are increased by air and reduced by fluids and cells), real-time monitoring of pulmonary ventilation changes at the bedside is achieved by computer imaging technology (3, 4). Bickenbach et al. showed EIT enables monitoring of regional ventilation distribution during SBTs and is suitable to estimate whether an SBT probably will be beneficial for an individual patient (5). Recent clinical evidence has shown that the different etiologies of acute respiratory failure (ARF) have different regional lung ventilation and perfusion characteristics, as measured by the saline contrast EIT method (6). Zhang et al. demonstrated that EIT could identify the diverse effects of a high-flow nasal cannula on regional lung ventilation in post-extubation situations, which might be helpful to guide HFNC therapy in clinical practice (7). EIT is expected to become a new detection method for aiding mechanical ventilation withdrawal (5, 8). Accordingly, the purpose of this study was to evaluate the predictive value of the EIT in patients with delayed ventilator withdrawal after upper abdominal surgery.

## Materials and Methods

### Patients

This retrospective study included intensive care unit patients who had undergone upper abdominal surgery between January 1, 2018 and August 31, 2019. They were stratified into successful weaning (group S) and weaning failure groups (group F). The inclusion criteria were as follows: (1) age > 18 years, (2) postoperative mechanical ventilation >24 h, (3) SBT performed before weaning, and (4) EIT monitoring performed during weaning. The exclusion criteria were as follows: (1) poor EIT images unsuitable for data analysis and (2) currently pregnant. The Ethics Committee of Sichuan Provincial People's Hospital approved the study. We have obtained the consent of all patients.

### Weaning Process

An SBT was considered when the patient met the weaning criteria: (1) the primary disease causing respiratory failure was controlled; (2) partial pressure of oxygen (PaO_2_)/fraction of inspired oxygen (FiO_2_) ≥ 150 mmHg, with positive end-expiratory pressure ≤ 8 cm H_2_O]; (3) stable hemodynamic state [heart rate (HR) ≤ 140 bpm, 90 mmHg < systolic blood pressure <160 mmHg, and no vasoactive drugs]; (4) strong ability of spontaneous breathing and coughing; (5) body temperature <38°C; (6) no obvious respiratory acidosis [pH > 7.3, ${\text{HCO}}_{3}^{-}$ <30 mmol/L, or partial pressure of carbon dioxide (PaCO_2_) <45 mmH_2_O]; (7) hemoglobin level ≥ 80 g/L; and (8) good mental status. Before beginning the SBT, the ventilator settings were adjusted to the pressure support ventilation mode, and the parameter settings were as follows: pressure support = 8 cmH_2_O, FiO_2_ = 40%, positive end-expiratory pressure ≤ 5 cmH_2_O, and oxygen saturation (SpO_2_) maintained at ≥ 92%; the ventilation time in the whole pressure support ventilation mode was longer than 30 min. For patients then underwent the SBT, ventilator therapy was discontinued while endotracheal intubation was retained. Oxygen was administered through the tracheal tube with oxygen-enriched humidified air for 30 min at a rate of 2–4 L/min; the vital signs and SpO_2_ of the patients were monitored. Blood gas analysis was performed after 30 min. An SBT was classified as failed when any of the following conditions were met: (1) aggravation or recurrence of the primary disease (judged by a competent doctor); (2) respiratory rate ≥ 35 breaths/min, an increase of 50% from baseline, or ≤ 8 breaths/min; (3) hemodynamic instability (systolic blood pressure > 180 or <90 mmHg, or pulse > 140 beats/min); (4) basal HR or blood pressure change rate > 20% or FiO_2_ > 0.5, PaO_2_ <60 mmHg, or PaCO_2_ increase > 10 mmHg; (5) pH <7.32 or pH decrease > 0.07; and (6) anxiety, sweating, or irritability (judged by the doctor in charge). Patients who passed the SBT were extubated and administered oxygen using a nasal catheter or mask at a rate of 2–4 L/min. For patients who did not pass the SBT, the doctor chose the appropriate ventilation mode to continue mechanical ventilation according to the situation and recorded the weaning failure. Weaning failure was defined as follows: (1) failed SBT, (2) re-treatment with invasive or non-invasive ventilation within 48 h after extubation, and (3) death within 48 h after weaning. Acute Physiology and Chronic Health Evaluation II score, HR, mean arterial pressure (MAP), arterial blood gases index (pH, PaO_2_, PaCO_2_, and lactate levels), oxygenation index (PaO_2_/FiO_2_), and SpO_2_ were recorded before weaning on the same day. We collected age, sex, type of sugery, Acute Physiology Score II values, SBT's result. At the same time, the HR, respiratory rate, MAP, SpO_2_, PaO_2_, PaCO_2_, at 0, 5, 15, and 30 min of SBTs were collected in both study groups.

#### EIT Data Acquisition and Analysis

When the patient met the weaning criteria, a silicon 16-electrode EIT belt of proper size was placed around the patients' chest between the 4th and 6th ribs and connected to the EIT device (PulmoVista 500; Dräger Medical GmbH, Lübeck, Germany). EIT scans are represented by 32 × 32 color-coded matrix images. A low pass filter with a cutoff frequency of 50 min^−1^ was applied to exclude cardiac-related variations. During all study phases, EIT data were generated by application of electrical current at 25 kHz, recorded by an EIT device. EIT measurements were started at the beginning of SBT and obtained for 5 min at 0, 5, 15, and 30 min during SBT. The last 2 min of each record were analyzed. We recorded the SBT data at 0 min as the baseline value. With the generated tidal images, four horizontal layers from the ventral to the dorsal side were defined as regions of interest (ROI) and numbered from 1 to 4. Global inhomogeneity (GI) was measured as previously described (9, 10). The end-expiratory lung impedance (EELI) was defined as the average end-expiratory global impedance values of 10 consecutive breaths in SBT. With SBT at 0 min set as baseline, the change in end-expiratory lung impedance (ΔEELI) was defined as the difference between the EELI value at other time points (SBT at 5, 15, and 30 min) and the EELI value at baseline time (11, 12). We recorded the tidal volume of five subsequent breathing cycles at the last 1 min before SBT and calculated the average value as tidal volume (VT). ΔEELI/VT was defined as ΔEELI divided by VT.

The standard deviation of the regional ventilation delay index (RVDSD) was used to express regional ventilation distribution. The global impedance-time curve ΔZ(t) was as the sum of the impedance changes of all pixels. The RVDSD was assessed by EIT as previously described (13, 14). We calculated compliance win (CW) and compliance loss (CL), expressed as a percentage, from the EIT recordings (15). Compliance change percentage variation (|Δ(CW-CL)|) was defined as the difference between CW and CL regarding SBT at 5, 15, and 30 min compared to SBT at 0 min.

Regional distribution of ventilation measurements was recorded on the PulmoVista 500 during monitoring sessions. Each recording was transferred to a USB storage device. Data were downloaded from the USB storage device to a Windows (Microsoft, Redmond, Washington) based personal computer. The EIT data were analyzed offline using the EIT Data Analysis Tool 6.1 and 6.1.20 (Dräger Medical GmbH, Lübeck, Germany) and MATLAB 8.3 (The Mathworks, Natick, MA, USA).

### Statistical Analysis

Mean data were analyzed using SPSS software (version 22.0; IBM Corp., Armonk, NY, USA). Normally distributed data are presented as means ± standard deviations. Non-normally distributed data are presented as medians (interquartile intervals). Normally distributed and non-parametric continuous variables were compared using the Student's *t*-test and Mann–Whitney *U*-test, respectively. Basic clinical parameters were tested by the Wilcox non-parametric test. Repeated data were analyzed with a generalized estimation equation. The receiver operating characteristic curve was used to assess the accuracy of successful weaning. Cutoff values were obtained by calculating the Youden index, and the sensitivity and specificity were also determined. Considering the small sample size in this study, the positive and negative likelihood ratios were calculated using MedCalc software (version 19.0; MedCalc Software Ltd., Ostend, Belgium) to reduce the impact on sensitivity and specificity and evaluate the predictive value of each prediction index. Positive-predictive value, negative-predictive value, and diagnostic accuracy were also calculated. A *P* < 0.05 was considered statistically significant.

## Results

A total of 32 patients (22 men and 10 women) were included in this study. There were 23 patients in group S and 9 in group F. In group F, within 48 h after extubation, 5 patients received non-invasive ventilation only, 3 patients received invasive ventilation, and 1 patient was reintubated after failing to maintain with non-invasive ventilation. There were no significant differences in age, mechanical ventilation time, Acute Physiology and Chronic Health Evaluation II score, baseline HR, MAP, or values of arterial blood gases indices between the two groups (*P* > 0.05) for the SBT baseline (0 min) values (Table 1). However, there was a statistically significant difference in SpO_2_ between the two groups before the SBTs (*P* <0.05) (Table 1). At the end of the SBTs, there were no differences in HR, MAP, PaO_2_/FiO_2_, PaCO_2_, or pH between the two groups (Table 2).

**Table 1:** Baseline characteristics of patient is at SBT 0 min.

	***S* (*n* = 23)**	***F* (*n* = 9)**	***p***
Age (year)	58.48 ± 12.76	64.44 ± 15.13	0.31
Mechanical ventilation hours (h)	60.00 ± 45.91	72.00 ± 36.72	0.19
APACHE II	12.70 ± 4.54	12.33 ± 3.46	0.80
ASA index			
I, *n* (%)	16(69.5)	6(66.7)	1.00
≥II, *n* (%)	7(30.4)	3(33.3)	1.00
Type of surgery			
High surgical aggression, *n* (%)	12 (52.2)	4 (0.44)	1.00
Pancreaticodoudenectomy, *n* (%)	3(13.0)	1(11.1)	
Esophagectomy, *n* (%)	4(17.4)	2(22.2)	
Total gastrectomy, *n* (%)	5(21.7)	1(11.1)	
Intermediate surgical aggression	11 (47.8)	5 (55.5)	1.00
Total colectomy, *n* (%)	5(21.7)	2(22.2)	
Rectal resection, *n* (%)	2(8.7)	0(0)	
Major liver resection, *n* (%)	4(17.4)	3(33.3)	
Chronic disease			
Hypertension, *n* (%)	6(26)	2(22.2)	
Diabetes, *n* (%)	5(21.7)	1(11.1)	
Chronic kidney disease, *n* (%)	2(8.7)	0	
Chronic liver disease, *n* (%)	3(13.0)	1(11.1)	
Chronic obstructive pulmonary disease, *n* (%)	4(17.4)	1(11.1)	
Smoking status			
Never, *n* (%)	3(13.0)	1(11.1)	
Former, *n* (%)	16(69.5)	6(66.7)	
Current, *n* (%)	4(17.4)	2 (22.2)	
RSBI	90.13 ± 14.18	109.11 ± 13.77	0.006
P0.1	3.10 ± 0.82	3.56 ± 1.00	0.224
HR (times/min)	87.17 ± 15.50	90.11 ± 18.41	0.753
RR (times/min)	18.17 ± 2.79	20.33 ± 1.94	0.107
MAP (mmHg)	82.13 ± 11.76	85.41 ± 16.04	0.378
SpO_2_ (%)	99.52 ± 1.16	97.67 ± 2.12	0.002
pH	7.42 ± 0.07	7.43 ± 0.07	0.737
PaO_2_ (mmHg)	112.58 ± 31.10	120.07 ± 51.47	0.917
PaCO_2_ (mmHg)	39.40 ± 4.88	37.41 ± 6.72	0.450
PaO_2_/FiO_2_	281.45 ± 77.76	300.17 ± 128.68	0.917
Lac (mmol/L)	1.65 ± 0.68	1.51 ± 0.76	0.571

*APACHE II, acute physiology and chronic health evaluation II; PaCO_2_, arterial partial pressure of carbon dioxide; RSBI, rapid shallow breathing index; P0.1, occlusion pressure at 100 ms; HR, heart rate; RR, respiratory rate; MAP, Mean arterial pressure; SpO_2_, saturation of pulse O_2_; PaO_2_, arterial partial pressure of oxygen; PaCO_2_, arterial partial pressure of carbon dioxide; Lac, lactic acid; ASA, American Society of Anesthesiologists*.

**Table 2:** Patient's characteristics and arterial blood gases in patients at the end of SBT.

	***S* (*n* = 23)**	***F* (*n* = 9)**	***p***
HR (times/min)	88.91 ± 13.71	95.89 ± 18.14	0.25
RR (times/min)	19.82 ± 2.91	20.22 ± 3.93	0.76
MAP (mmHg)	83.4 ± 13.15	85.56 ± 12.89	0.63
SpO_2_ (%)	99.13 ± 1.91	97.33 ± 3.57	0.18
pH	7.41 ± 0.05	7.42 ± 0.06	0.96
PaO_2_ (mmHg)	110.76 ± 30.74	93.44 ± 38.39	0.19
PaCO_2_ (mmHg)	42.07 ± 5.38	45.06 ± 14.13	0.55
PaO_2_/FiO	285.3 ± 74.90	259.10 ± 92.00	0.41
Lac (mmol/L)	1.61 ± 0.58	1.80 ± 1.15	0.63

*SBT, Spontaneous Breathing Trial; HR, Heart rate; RR, respiratory rate; MAP, mean arterial pressure; SpO_2_, Pulse oxygen saturation; PaO_2_, arterial partial pressure of oxygen; PaCO_2_, arterial partial pressure of carbon dioxide; PaO_2_/FiO_2_, arterial partial pressure to inspired fraction of oxygen ratio; Lac, lactic acid*.

Generalized estimation equation analysis showed that ΔEELI/VT was lower in group F than in group S (*P* = 0.01) and was not associated with the measurement time (*P* = 0.17). The GI, RVDSD, and |Δ(CW-CL)| were higher in group F than in group S (*P* = 0.00, *P* = 0.00, *P* = 0.00) and were associated with the measurement time (*P* = 0.00; *P* = 0.00, *P* = 0.01). The value of ΔEELI/VT, GI, RVDSD, |Δ(CW-CL) | were show in Table 3.

**Table 3:** EIT data in patients with extubation success and failure.

	**SBT 0**	**SBT 5**	**SBT 15**	**SBT 30**
**ΔEELI/VT**				
Extubation success (*n* = 23)	0	−0.81 ± 0.26	−0.81 ± 0.27	−0.81 ± 0.26
Extubation failure (*n* = 9)	0	−1.02 ± 0.19	−1.13 ± 0.20	−1.10 ± 0.19
*P*-value of extubation success vs. failure		0.03	0.00	0.01
**RVD SD**				
Extubation success (*n* = 23)	9.35 ± 1.62	11.66 ± 2.59	11.60 ± 1.64	13.12 ± 2.22
Extubation failure (*n* = 9)	10.72 ± 2.08	13.44 ± 1.94	14.88 ± 3.07	16.73 ± 1.06
*P*-value of extubation success vs. failure	0.29	0.07	0.00	0.00
**Δ(CW-CL)**				
Extubation success (*n* = 23)	0	2.72 ± 2.32	3.60 ± 3.82	2.38 ± 2.45
Extubation failure (*n* = 9)	0	16.78 ± 8.22	21.10 ± 9.21	11.38 ± 6.57
*P*-value of extubation success vs. failure		0.00	0.00	0.00
**GI**				
Extubation success (*n* = 23)	0.51 ± 0.14	0.67 ± 0.18	0.71 ± 0.18	0.76 ± 0.19
Extubation failure (*n* = 9)	0.65 ± 0.15	0.70 ± 0.14	1.13 ± 0.24	1.14 ± 0.24
*P*-value of extubation success vs. failure	0.02	0.60	0.00	0.00

*ΔEELI/Vt, change from baseline of the end-expiratory lung impedance/the tidal volume in percentage; RVDSD, standard deviation of regional ventilation delay index; Δ(CW-CL), compliance change percentage variation; GI, global inhomogeneity*.

**Figure 1:**
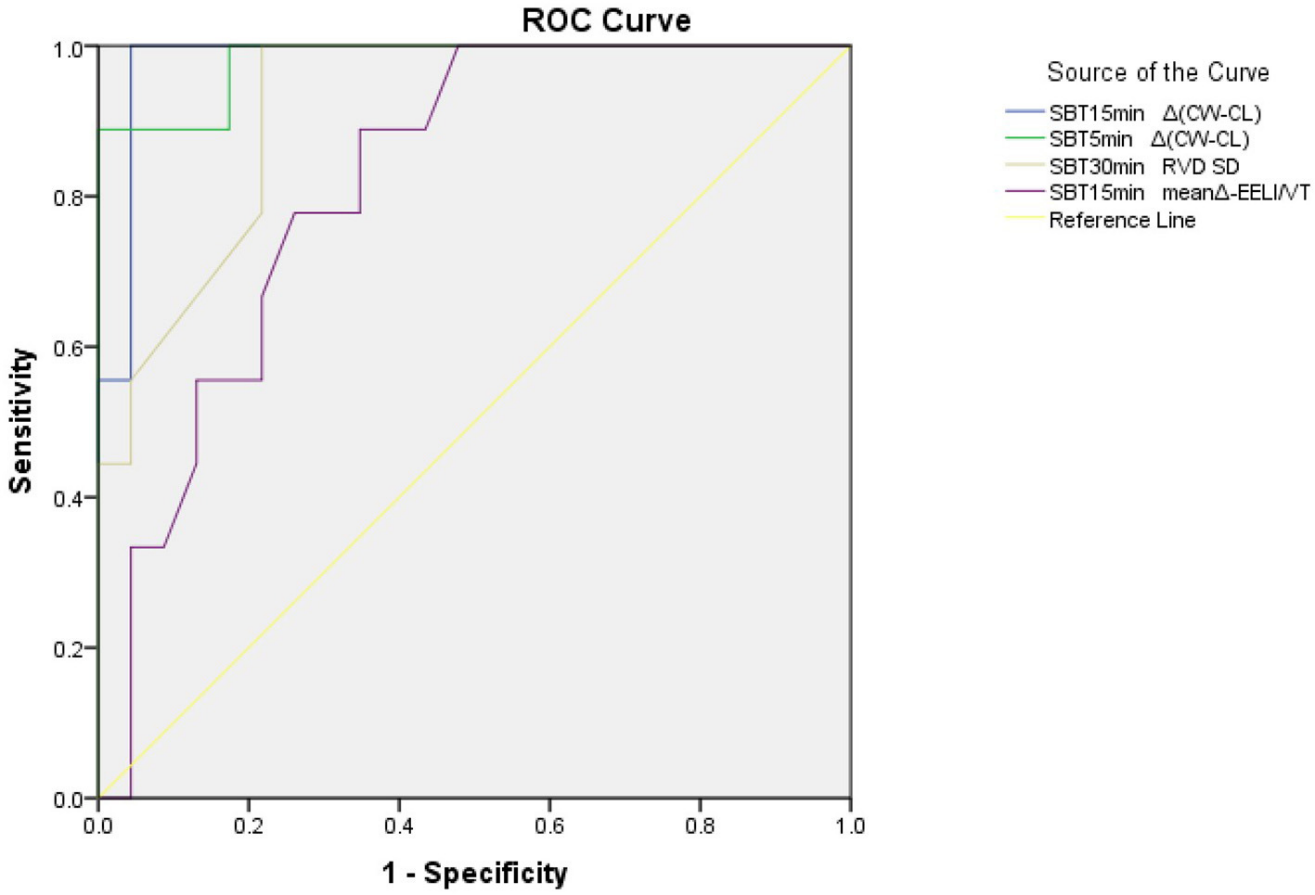
A ROC of prognostic variables for patients. ΔEELI/Vt, change from baseline of the end-expiratory lung impedance/the tidal volume in percentage; RVDSD, standard deviation of regional ventilation delay index; Δ(CW-CL), compliance change percentage variation; GI, global inhomogeneity.

The areas under the curve (AUCs) of the ΔEELI/VT of SBTs at 5, 15, and 30 min were 0.742,0.819,0.816, respectively. The cutoff value of the ΔEELI/VT of SBTs was −0.89 at SBT15 min, and the sensitivity and specificity for predicting weaning successful were 88.89 and 65.22%, respectively. The AUC of the |Δ(CW-CL)| of SBTs at 5 min was 0.981. The cutoff value of the |Δ(CW-CL)| of SBTs at 5 min was more than 7.7; the sensitivity and specificity for predicting weaning failure was 88.89 and 100%, respectively, the positive-predictive value was 100%, and the accuracy was 0.89. The AUC of the |Δ(CW-CL)| of SBTs at 15 min was 0.981; the cutoff value of the |Δ(CW-CL)| of SBTs at 15 min was more than 7.4; the sensitivity and specificity for predicting weaning failure was 100 and 95.65%, respectively; the positive-predictive value was 90%; and the accuracy was 0.96. The AUC of RVDSD of SBTs at 30 min was 0.918; the cutoff value of RVDSD of SBTs at 30 min was more than 15.7; the sensitivity and specificity for predicting weaning failure was 100 and 78.26%, respectively; the positive-predictive value was 64.3%; and the accuracy was 0.78 (Figure 1).

## Discussion

In this study, we found that compared to group S, ΔEELI/VT was lower, and the GI, RVDSD, and |Δ(CW-CL)| were higher in group F. In the early stage of SBT, the |Δ(CW-CL)|, as well as ΔEELI/VT, have a good predictive value of the failure rate of weaning, while the RVDSD has a good predictive value of the failure rate of weaning in the end stage of SBT. The present findings suggest that EIT, a new method to evaluate pulmonary gas distribution, might be a potential tool that could aid patients undergoing upper abdominal surgery with ventilatory weaning.

Because of the pain and repeated diaphragmatic stimulation experienced intraoperatively, pulmonary complications in patients are significantly higher after upper abdominal surgery than after cardiac surgery (16). In addition, due to the effects of anesthesia, mechanical ventilation, and changes in intrathoracic and abdominal pressure, the occurrence of early postoperative atelectasis is almost inevitable. Notably, early ventilator withdrawal can effectively prevent the occurrence of ventilator-associated pneumonia (17). Accordingly, for patients with mechanical ventilation after upper abdominal surgery, clinicians need to actively find an appropriate time to speed up the process of ventilator withdrawal to reduce the delayed extubation. Therefore, an ideal predictor of weaning is urgently needed in clinical settings.

EIT has been a major breakthrough in the field of biomedical engineering in recent decades. Because the electrical impedances of tissues, gas, and liquid are different, the computer collects the electrical impedance information and reconstructs it into an image. Accordingly, an image of the gas distribution in the process of lung ventilation is obtained. During mechanical breath, at the initial stage of inspiration, the increase in airway pressure causes the gas to enter the gravity-independent zone, while in the later stage of inspiration, the continuously inhaled gas is gradually distributed to the gravity-dependent zone. The context is more complicated during spontaneous breath. Therefore, alveolar ventilation has three characteristics: time distribution, spatial distribution, and global distribution. To date, EIT is the only method that can monitor the entire pathophysiological process of alveolar ventilation in real-time and dynamically at the bedside. Theoretically, it can reflect the whole lung and local conditions. In this study, the ΔEELI/VT, RVDSD, |Δ(CW-CL)|, and GI were used to evaluate weaning.

EIT can be used to monitor the compliance of local alveoli to increase or decrease and is not affected by positive pressure ventilation, which is more convenient for patients undergoing SBT. ΔEELI represents the variation in end-expiratory lung impedance during the SBT. The decrease in ΔEELI suggested that there may be part of the alveoli collapsed or the VT decreased. Because the EELI are infected by VT, we chose to use VT to reduce this heterogeneity. ΔEELI/VT reflected the change in end-expiratory lung impedance per unit volume at the different SBT time points, which may guide patients with ventilatory weaning. Our study found that compared to patients under successful weaning, patients experiencing weaning failure were characterized by greater ΔEELI/VT loss during STB, probably because alveolar collapse occurred more in group F, thereby resulting in decreased end-expiratory volume and reduced EELI. Longhini et al. (18) and Mauri et al. (19) also showed that ΔEELI was significantly higher in patients with successful weaning, consistent with our findings. RVDSD can show the time delay of the pulmonary ventilation area, which can be used as a parameter to reflect the time distribution of pulmonary ventilation. Muders et al. (13) observed a positive correlation between RVDSD and computed tomography imaging. The greater areas of the collapsed alveoli, the greater the value of RVDSD. In addition, Muders et al. found that due to increased alveolar collapse and overall lung heterogeneity, patients with failed weaning might not be able to maintain adequate alveolar ventilation. In our study, we found that the RVDSD values of SBTs in group F were higher than those in group S. Moreover, at the end of SBT, using the cutoff of 15.7, the AUC for predicting failure withdrawal was 0.91, the sensitivity was 100%, and the specificity was 78%, suggesting that the RVDSD maybe a good indicator at the end of SBT. GI—the proportion of the sum of the impedance value of the whole lung pixel and the average impedance value to the sum of the pixel impedance value in the whole lung area—is a parameter used to evaluate the ventilation state of the whole lung. In our study, the GI index of the two groups increased with increases SBT time, and the GI index of group F increased more significantly than that of group S. It is likely that during T-tube ventilation, part of the alveoli collapsed due to the lack of positive pressure ventilation. In group F, the adjustment of spontaneous respiration could not maintain the normal opening of alveoli, especially near the diaphragm, thereby leading to an increase in uneven gas distribution in the lungs and a decrease in alveolar ventilation, which resulted in weaning failure. Pulmonary compliance is important in the regulation of pulmonary ventilation. Previous studies found that poor pulmonary compliance during SBT was associated with extubation failure, with poor accuracy for predicting weaning success, especially when weaning was based on the value of decreased compliance alone (20, 21). Our study found that the |Δ(CW-CL)| was significantly higher in patients with failed weaning than in those with successful weaning. Especially in the early time of SBT, the value of |Δ(CW-CL)| with failure group was more obviously. It may be related to excessive or weak spontaneous breathing. Excessive spontaneous breathing can easily lead to ventilator fatigue and self-induced lung damage, while weak spontaneous breathing may lead to carbon dioxide retention; both excessive and weak spontaneous breathing lead to failed ventilator weaning. In our study, we considered that |Δ(CW-CL)| may be a good prediction parameter in the early stages of SBT.

Our study has some limitations. First, we did not monitor the increase in intra-abdominal pressure, which may have affected pulmonary compliance, diaphragmatic movement, and transpulmonary pressure, thus affecting the patient's spontaneous breathing. Notably, the phenomenon of increased abdominal pressure was not recorded in any patient after physical examination. Second, this study was a retrospective study, and the weaning method used was an SBT; therefore, it was impossible to compare the related guiding indicators of EIT and the predictive values of SBTs for weaning. These limitations need to be addressed in future clinical studies.

## Conclusions

This study found that GI, the RVDSD, and the |Δ(CW-CL)| of SBTs were higher, while ΔEELI/VT was lower, in group F than in group S. In the early stage of SBT, the |Δ(CW-CL)|, as well as ΔEELI/VT, may have good predictive values for weaning failure rate, while the RVDSD may have a good predictive value for weaning failure rate in the end stage of SBT. These findings suggest that EIT may became a predictor of ventilator weaning in patients undergoing upper abdominal surgery.

## Data Availability Statement

The original contributions presented in the study are included in the article/supplementary material, further inquiries can be directed to the corresponding authors.

## Ethics Statement

The studies involving human participants were reviewed and approved by the Ethics Committee of Sichuan Provincial People's Hospital affiliated to Sichuan Provincial People's Hospital. Written informed consent for participation was not required for this study in accordance with the national legislation and the institutional requirements.

## Author Contributions

JL and FZ wrote the paper. RL, YLa, and FY were responsible for the program implementation. FZ and XL were responsible for data analysis. YR, YLe, and XH were responsible for data collection. GZ were responsible for evolution of overarching research goals and aims, management and coordination responsibility for the research activity planning and execution. All authors contributed to the study design, and read and approved the submitted version of the manuscript.

## Funding

The authors declare that this study received funding from the Science and Technology Support Program of Sichuan Province, China (Grant Numbers 2017SZ0138 and 20ZDYF1870) and Funding of Provincial People's Hospital Program (Grant Number 2020ZX02). The funders were not involved in the study design, collection, analysis, interpretation of data, the writing of this article or the decision to submit it for publication.

## Conflict of Interest

The authors declare that the research was conducted in the absence of any commercial or financial relationships that could be construed as a potential conflict of interest.

## Publisher's Note

All claims expressed in this article are solely those of the authors and do not necessarily represent those of their affiliated organizations, or those of the publisher, the editors and the reviewers. Any product that may be evaluated in this article, or claim that may be made by its manufacturer, is not guaranteed or endorsed by the publisher.
